# The autophagy scaffold protein ALFY is critical for the granulocytic differentiation of AML cells

**DOI:** 10.1038/s41598-017-12734-4

**Published:** 2017-10-11

**Authors:** Anna M. Schläfli, Pauline Isakson, E. Garattini, Anne Simonsen, Mario P. Tschan

**Affiliations:** 1 Division of Experimental Pathology, Institute of Pathology, University of Bern, Bern, Switzerland; 2 Graduate School for Cellular and Biomedical Sciences, University of Bern, Bern, Switzerland; 3 Clinical immunology & transfusion medicine, Sahlgrenska University Hospital, Gothenburg, Sweden; 4 Laboratory of Molecular Biology, Istituto di Ricerche Farmacologiche ‘Mario Negri’, Milano, Italy; 5 Department of Molecular Medicine, Institute for Basic Medical Sciences, University of Oslo, Oslo, Norway

## Abstract

Acute myeloid leukemia (AML) is a malignancy of myeloid progenitor cells that are blocked in differentiation. Acute promyelocytic leukemia (APL) is a rare form of AML, which generally presents with a t(15;17) translocation causing expression of the fusion protein PML-RARA. Pharmacological doses of all-trans retinoic acid (ATRA) induce granulocytic differentiation of APL cells leading to cure rates of >80% if combined with conventional chemotherapy. Autophagy is a lysosomal degradation pathway for the removal of cytoplasmic content and recycling of macromolecules. ATRA induces autophagy in ATRA-sensitive AML and APL cells and autophagy inhibition attenuates ATRA-triggered differentiation. In this study, we aimed at identifying if the autophagy-linked FYVE-domain containing protein (ALFY/WDFY3) is involved in autophagic degradation of protein aggregates contributes to ATRA therapy-induced autophagy. We found that ALFY mRNA levels increase significantly during the course of ATRA-induced differentiation of APL and AML cell lines. Importantly ALFY depletion impairs ATRA-triggered granulocytic differentiation of these cells. In agreement with its function in aggrephagy, knockdown of ALFY results in reduced ATRA-induced proteolysis. Our data further suggest that PML-RARα is an autophagy substrate degraded with the help of ALFY. In summary, we present a crucial role for ALFY in retinoid triggered maturation of AML cells.

## Introduction

Acute myeloid leukemia (AML) is a cancer of the myeloid compartment characterized by a block of leukemic cells at different levels along the myeloid differentiation process. This leads to clonal expansion of immature cells in the bone marrow, blood and other tissues with a concomitant decrease in normal and functional myeloid cells^1^. At the molecular level AML is a very heterogeneous disease as indicated by the large number of chromosomal alterations and mutations found^2^. A distinct subtype of AML is acute promyelocytic leukemia (APL), which is associated with a translocation involving the retinoic acid receptor A (*RARA*) on chromosome 17. The most commonly found fusion involves the promyelocytic leukemia protein (*PML*) and *RARA* ^3^. APL is a very aggressive form of AML with a rapid disease progression. Currently, APL is a highly curable disease thanks to established therapeutic protocols based on the use of all-trans retinoic acid (ATRA) in combination with anthracyclines and, more recently, arsenic trioxide^4,5^^,^.

The classical mechanism believed to underlay the molecular pathology of APL is based on the contention that PML-RARα exerts a dominant negative effect on the transcription of RARα target genes, which impairs their responsiveness to physiological concentrations of ATRA^6^. In addition the DNA binding sites of PML-RARα and the key myeloid transcription factor, PU.1, are generally overlapping. This observation has led to the idea that PML-RARα represses transcriptional activation of PU.1 target genes^7^. Finally, PML-RARα has an increased repertoire of DNA binding sites as compared to RARα^8^, which suggests gain-of-function properties of the fusion protein. Pharmacological doses of ATRA overcome the PML-RARα mediated differentiation block of APL cells. Although ATRA leads to high rates of complete remission due to the induction of granulocytic differentiation and subsequent cell death, the available clinical data indicate that ATRA monotherapy invariably leads to relapse of the disease. This has led to the idea that targeting of leukemia initiating cells (LICs) by ATRA is inefficient^9^. With respect to this last point, it must be emphasized that degradation of PML-RARα is deemed to be a key event in the elimination of LICs, which is the pre-requisite for a long-term cure of APL^10^. Several pathways are implicated in the ATRA-dependent degradation of PML-RARα, such as direct targeting by caspases and proteosomal degradation^11,12^. A role for autophagy in ATRA-induced degradation of PML-RARα has recently been established^13,14^. Indeed, inhibition of autophagy by pharmacological agents reduces ATRA-induced granulocytic differentiation of APL cells^13^. Similarly to PML-RARα, leukemic oncofusion proteins such as BCR-ABL and FLT3-ITD are subjected to autophagic degradation during targeted therapies^15^.

Autophagy is a process that leads to the degradation of cytoplasmic constituents in the lysosomal compartment. Depending on how the cargo enters the lysosome, three types of autophagy can be distinguished: Macroautophagy, chaperone-mediate autophagy and microphagy^16^. Macroautophagy (hereafter referred to as autophagy) involves the formation of a double membrane structure, the so called autophagosome, which finally fuses with the lysosome to degrade its content. The process of autophagy is executed by numerous autophagy related genes (ATGs), whose encoded proteins generate the autophagosome in an orchestrated manner^17^. Depending on the substrate macroautophagy can be further subdivided in distinct sub-processes. For instance, aggrephagy is the selective removal of aggregated proteins, which tend to accumulate in the cytoplasm and disturb the proper cellular function^18^. The principle of selective degradation by autophagy relies on the presence of a ligand, which specifically targets the cargo for degradation. Recognition of the ligand by an autophagy receptor enables interaction with the autophagy machinery and mediates cargo degradation. Autophagy scaffold proteins assist autophagy receptor proteins and serve as platforms to facilitate the interaction between the receptor targeted cargo and the autophagy machinery^19^. A well-known autophagy receptor is p62, which mediates the degradation of various ubiquitinated cargos including damaged mitochondria, protein aggregates and microbes^20^. Interestingly, p62 supports autophagy-dependent PML-RARα degradation^14^.

Autophagy-linked FYVE-domain containing protein (ALFY/WDFY3) is an autophagy scaffold protein and was originally identified as a PI3P interacting protein localizing to autophagic membranes^21^. Upon autophagy-induction, ALFY translocates from the nucleus to the cytoplasm where it assists p62 in the autophagy-dependent degradation of ubiqutinated protein aggregates in neurodegenerative diseases^22,23^. ALFY interacts with ATG5 in a protein complex consisting of ATG12, ATG5 and ATG16L^24^, supporting the notion that ALFY is an autophagy scaffold protein^25^. In line with this hypothesis, Lystad *et al*.^26^ identified a LC3-interacting region (LIR) in ALFY. The LIR is likely to mediate the interactions with different ATG8 family members. All this prompted us to investigate the role of ALFY in myeloid differentiation and aggrephagy.

## Results

### ALFY is upregulated during ATRA-induced differentiation of AML cells

**Figure 1:**
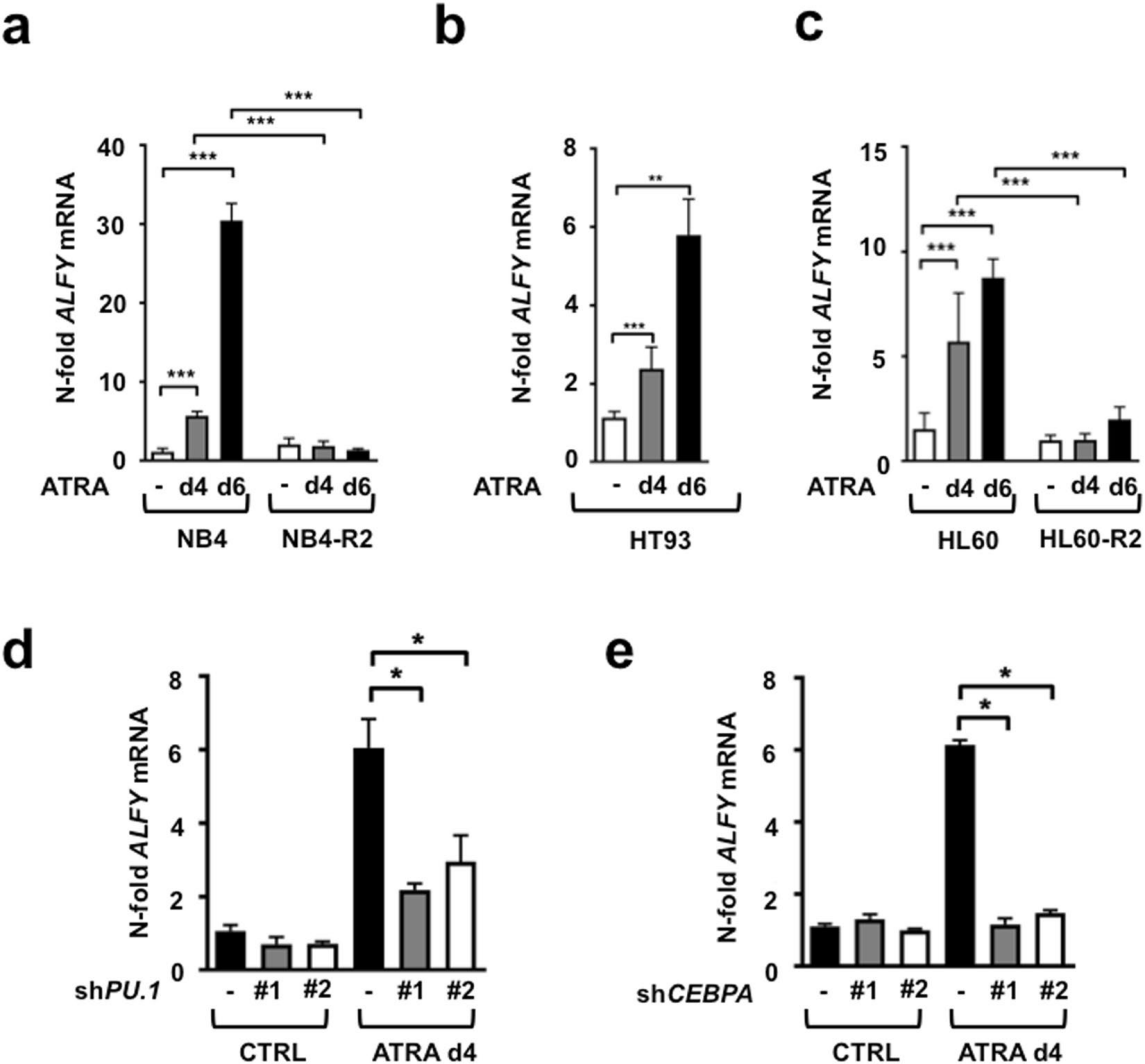
ALFY is upregulated during ATRA-induced differentiation of AML cells. (**a**–**c**) NB4, NB4-R2 and HT93 APL cells as well as the non-APL HL60 cell line and its ATRA-resistant clone HL60-R2 were treated with 1 µM ATRA for 4 and 6 days. *ALFY* mRNA levels were determined by qPCR. Raw Ct values were normalized to *HMBS* and to the untreated control of day 4. Standard deviations are shown for at least three independent experiments. (**d**–**e**) NB4 cells were transduced with two shRNAs (#1 and #2) targeting different regions of the *PU.1* or *CEBPA* mRNA or a non-targeting control (CTRL). After treatment with 1 µM of ATRA for 4 days *ALFY* mRNA levels were determined by qPCR and normalized as described in A.

In order to establish a role for ALFY in the granulocytic differentiation of AML cells, we exposed NB4 and HT93 APL as well as HL60 AML cells to ATRA for different amounts of time. In NB4 cells, ATRA exposure led to a 5.7- and 30.6-fold up-regulation of *ALFY* mRNA levels at day 4 and 6, respectively (Fig. 1a). A similar *ALFY* mRNA induction was observed in ATRA treated HT93 (Fig. 1b) and HL60 (Fig. 1c) cells. To support the idea that ATRA–triggered induction of *ALFY* mRNA is dependent on granulocytic differentiation, similar experiments were performed in the ATRA-resistant sub-clones NB4-R2 and HL60-R2. In both cell lines, no significant modification in the levels of *ALFY* mRNA was observed (Fig. 1a and c). Taken together, our data indicate that *ALFY* mRNA is upregulated only in AML cells, which undergo granulocytic differentiation upon exposure to ATRA. To evaluate whether ALFY is functionally involved in APL differentiation, we inhibited granulocyte maturation by knocking down the key myeloid transcription factors, PU.1 and CEBPα^27^. Knock-down efficiencies are shown in supplementary Figure 1. As expected, inhibition of these transcription factors resulted in reduced levels of differentiation, as indicated by lower mRNA levels of the well-established granulocyte differentiation marker *CEBPE* (Supplementary Figure 1). These effects were accompanied by a significant impairment of ALFY upregulation (Fig. 1d,e). The results are consistent with the notion that ALFY mRNA levels are upregulated in a PU.1- and CEBPα-dependent manner during APL cell differentiation.

### ALFY is down-regulated in primary AML and increases during normal neutrophil differentiation

**Figure 2:**
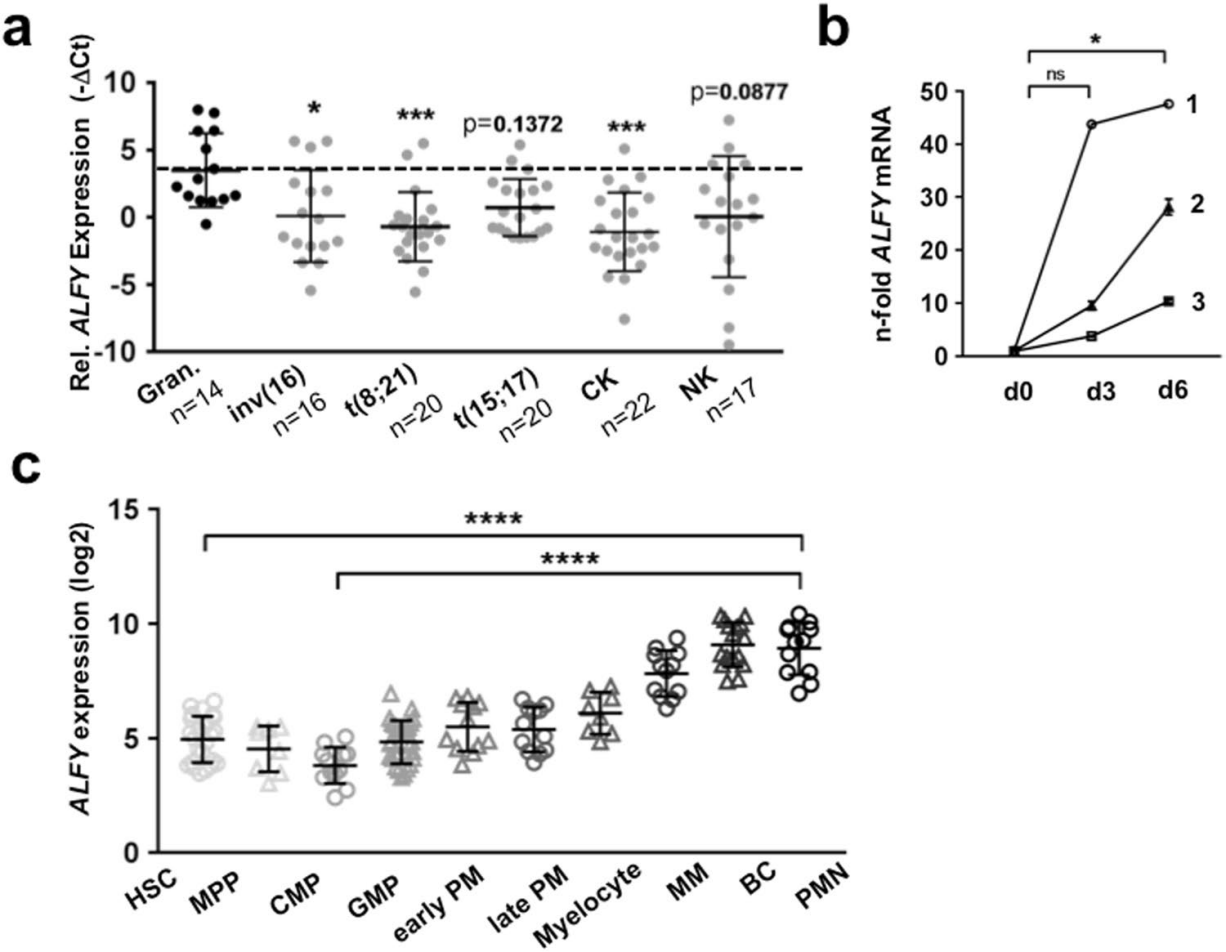
ALFY is down-regulated in primary AML and upregulated during normal neutrophil differentiation. **(a**) RNA was isolated from 95 patients with primary AML of FAB subtype M0-M4 and qPCR analysis of *ALFY* mRNA was performed using the TaqMan Low-Density Array (TLDA). Raw Ct values were normalized to *HMBS* and *ABL1* (−∆Ct). −∆Ct values of patient samples harbouring different chromosomal aberrations are shown and compared to the −∆Ct values of granulocytes from healthy donors (Gran.). CK = complex karyotype, NK = normal karyotype. Statistical test: Kruskall-Wallis test followed by corrected Dunn’s post hoc test for multiple comparison. *P < 0.05, **P ≤ 0.01, ***P ≤ 0.001. (**b**) CD34^+^ cells from three different donors (1–3) were *in vitro* differentiated towards granulocytes using G-CSF. *ALFY* transcript levels were measured at day 0 (d0), day 3 (d3) and day 6 (d6) using qPCR and normalized to *HMBS*. Friedman test followed by corrected Dunn’s post hoc test for multiple comparison was applied. * p < 0.05. (**c**) The panel displays the results obtained from the “normal haematopoiesis with AMLs” dataset contained in the bloodspot database. Please refer to the website for details on the dataset characteristics. HSC: hematopoietic stem cells, MPP: multipotent progenitors, CMP: common myeloid progenitor, GMP: granulocyte monocyte progenitor, PM: promyelocyte, MM: metamyelocyte, BC: band cell, PMN: polymorph nuclear cells. Kruskall-Wallis test followed by corrected Dunn’s post hoc test for multiple comparison was applied. **** p ≤ 0.0001.

Since *ALFY* expression is significantly induced during granulocytic differentiation of AML-derived cell lines, we compared *ALFY* mRNA levels in AML patients (FAB M0-M4) and granulocytes from healthy donors. The patient characteristics are listed in supplementary Table 1. In line with our findings that ALFY is upregulated in differentiated AML cells, we found significantly lower levels of *ALFY* in immature blast cells of primary AML patients when compared to mature neutrophils from healthy donors (Fig. 2a). Accordingly, *ALFY* transcript levels gradually increased during neutrophil differentiation of CD34^+^ from three healthy donors upon G-CSF treatment (Fig. 2b). In fact, we found a significant upregulation of *ALFY* mRNA on day 6 of G-CSF stimulation. These data were confirmed by an analysis performed on publicly available data contained in the bloodspot database^28^ (Fig. 2c). Taken together, our results suggest an involvement of ALFY not only in ATRA-triggered differentiation of AML cells but also in normal granulocytic maturation.

### ALFY is targeted by the oncogenic microRNA miR-181b

**Figure 3:**
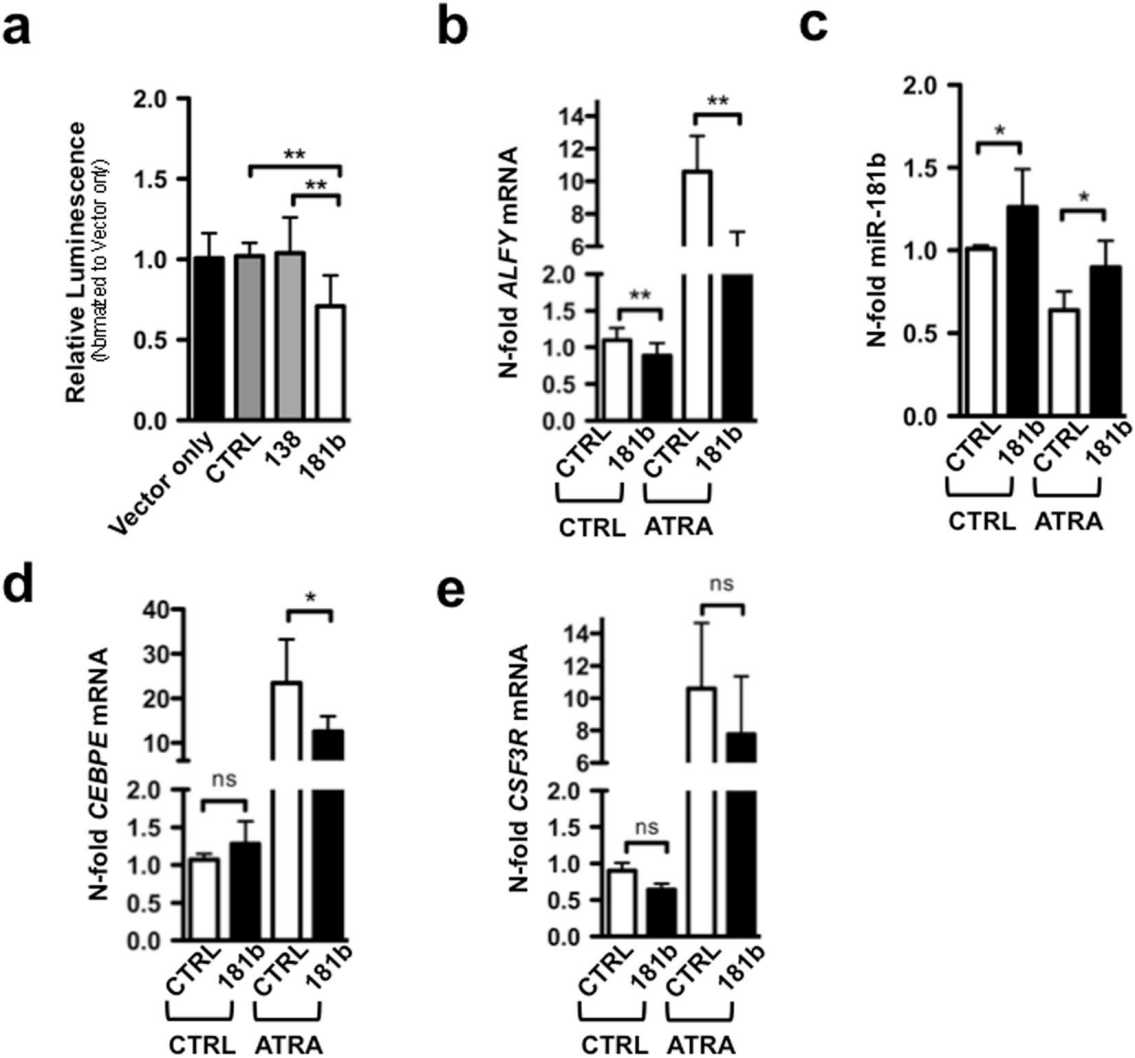
Oncogenic microRNA-181b is a negative regulator of ALFY mRNA in APL cell. (**a**) H1299 cells were seeded, transfected with a vector expressing a firefly luciferase under the control of the *ALFY* 3′UTR or together with a scrambled sequence (CTRL), miR-138 (138) or miR-181b (181). 24-hours later, luminescence was measured and the results are shown as n-fold regulation compared to the vector-only transfected cells. (**b**–**d**) NB4 cells were transduced with lentiviral vectors to over-express miR-181b (181) or a scrambled sequence (CTRL). Total RNA was isolated at day 4 of ATRA treatment. Values were normalized for the expression of the *SNORA66B* and *SNORA66* reference genes and are shown as n-fold regulation to untreated control transduced (CTRL) cells. In parallel, qPCR analyses of *ALFY*, *CEBPE* and *CSF3R* were performed. Standard deviations are shown for two independent experiments. Mann-Whitney-U test: *P < 0.05, **P ≤ 0.01.

To gather information as to the molecular mechanisms underlying ALFY mRNA induction by ATRA, we used the miRWalk prediction database^29^ and identified members of the miR-181 family as potential *ALFY* 3′UTR targeting miRNAs. Since miR-181b is over expressed in promyelocytes from APL patients cells and downregulated during normal and ATRA treated granulocyte development^30,31^, we focused our attention on this miRNA. Indeed, miR-181b specifically inhibited an *ALFY* 3′UTR reporter construct. In contrast, miR-138, which is not predicted to bind to *ALFY* 3′UTR, or a scrambled sequence, did not exert any effect on the reporter construct (Fig. 3a). Importantly, ectopic expression of miR-181b caused a significant down-regulation of the basal *ALFY* mRNA levels in NB4 cells grown under control conditions. The effect was more pronounced in NB4 cells exposed to ATRA (Fig. 3b). In ATRA-treated and miR-181b over-expressing NB4 cells, the down-regulation of *ALFY* mRNA was accompanied by reduced neutrophil differentiation as shown by a significant reduction in the amounts of mRNA coding for *CEBPE* (Fig. 3d). In the same experimental conditions we observed a trend towards a decrease in the levels of the *CSF3R* mRNA, coding for another granulocytic marker (Fig. 3e). Our results are consistent with the idea that miR-181b is a new negative regulator of *ALFY* mRNA during granulocytic differentiation of NB4 cells.

### ALFY significantly contributes to retinoid-mediated differentiation of AML cells

**Figure 4:**
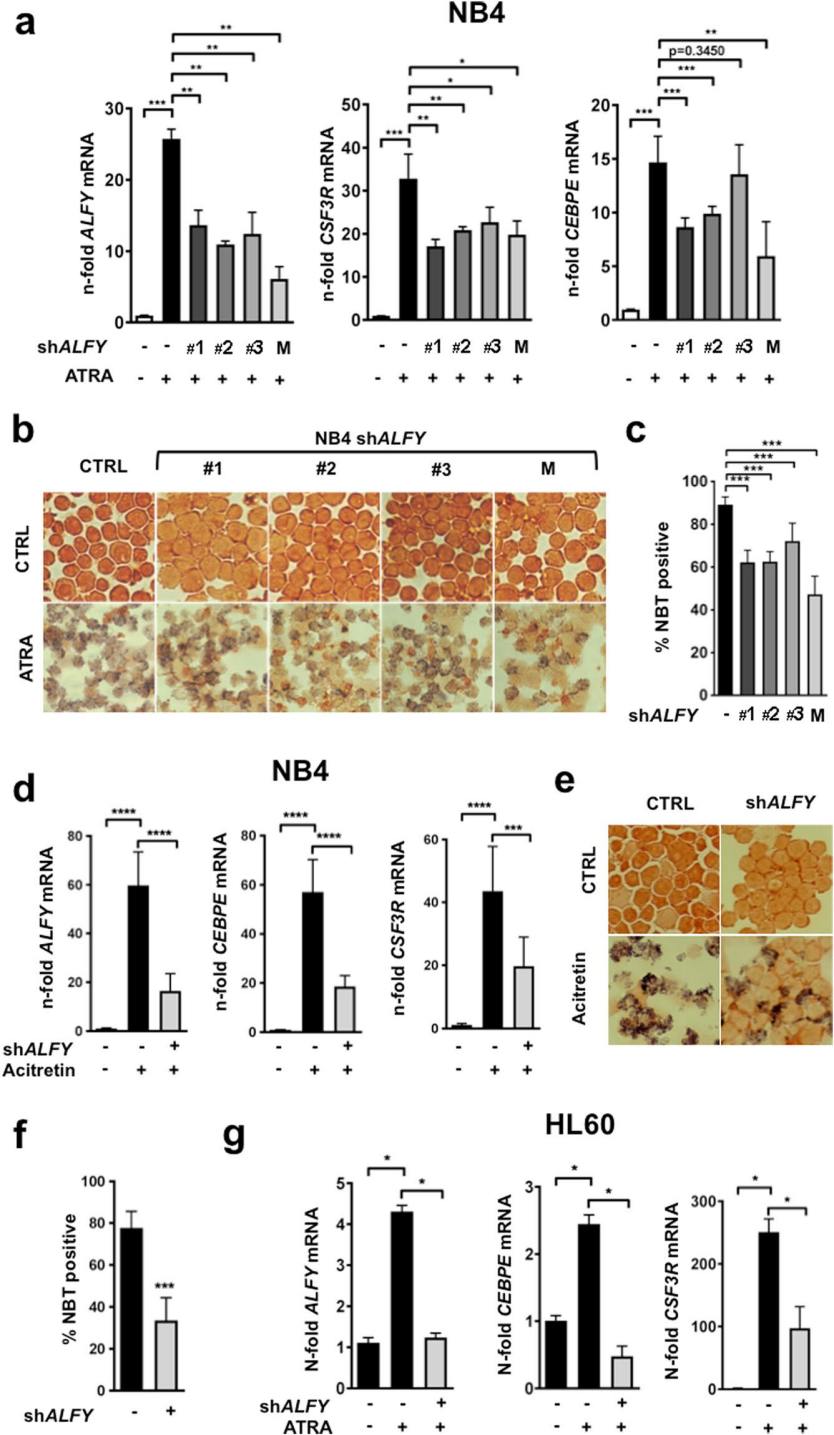
ALFY is crucial for retinoid-mediated differentiation of AML cells. Different NB4 or HL60 ALFY knock-down cell lines were generated by lentiviral transduction of shRNAs targeting the *ALFY* mRNA at three different sites (#1-#3) or a mixture of all three shRNAs (M). (**a**) NB4 shALFY cell lines or control-transduced cells (CTRL) were treated with 1 µM ATRA as indicated for 6 days before the assessment of granulocytic differentiation. Standard deviations are representative of three independent experiments. ALFY knockdown efficiency as well as *CSF3R* and *CEBPE* differentiation markers were determined by qPCR. Raw Ct values were normalized to *HMBS* and to the untreated control cell line using the 2^−∆∆CT^ method. (**b–c**) Neutrophil functionality and granulocytic differentiation were determined with the use of a nitroblue tetrazolium (NBT) reduction assay. NB4 control (CTRL) and shALFY cells were stimulated with phorbol myristate acetate (PMA) in the presence of NBT and subjected to the CytoSpin procedure. The percentage of NBT positive cells under ATRA or acitretin treatment was quantified. Differences in colour intensity were not considered. Mann-Whitney-U test: *P < 0.05, **P ≤ 0.01, ***P ≤ 0.001. (**d**) NB4 control (CTRL) or shALFY transduced cells were treated with 1 µM acitretin for 6 days and qPCR analyses were performed to measure *ALFY*, *CEBPE* and *GC3SFR* mRNAs. Raw Ct values were normalized to *HMBS* and to the untreated control of day 6 (∆∆Ct). Standard deviations were calculated on three independent experiments. (**e**-**f**) NBT assays were performed as described in Fig. 2. The percentage of positive cells was calculated from three independent experiments, comparing control and ALFY depleted cells under acitretin treatment at day 6 (**g**) HL60 control and shALFY transduced cells were treated with 1 µM ATRA and knockdown efficiency as well as differentiation was assessed by measuring *ALFY*, *CEBPE* and *CSF3R* mRNA expression. Normalization was performed as described in A. Mann-Whitney-U test: *P < 0.05, **P ≤ 0.01, ***P ≤ 0.001.

In order to test our hypothesis that ALFY is essential for granulocytic differentiation of APL cells, we generated four NB4-derived cell populations stably expressing three different small hairpin RNAs (shRNAs) constructs targeting *ALFY* (shALFY1–3) and a control shRNA construct (shC), using lentiviral vectors. The knock-down efficiencies of the three single shRNA constructs as well as the mixture of the corresponding shRNAs (shALFY M) are shown in Fig. 4a. Following exposure to ATRA for 6 days, the differentiation of ALFY-depleted NB4 cells was significantly reduced relative to the respective shC infected control cells, as indicated by the reduced expression of the *CEBPE* and *CSF3R* differentiation markers (Fig. 4a). We further established granulocytic differentiation using the nitro blue tetrazolium (NBT) reduction assay. As compared to the corresponding controls, ALFY knock-down caused a significant decrease in the number of NBT-positive cells following 6 days of ATRA exposure (Fig. 4b). Quantification of the NBT assay from 3 independent experiments indicates that approximately 90% of the control cell population was NBT-positive, while this positivity decreased to 50–70% following ALFY knock-down (Fig. 4c). The results were confirmed in the HT93 cell line (Supplementary Figure S2). To support a role for ALFY in the granulocytic maturation of APL cells, we repeated the experiments using the synthetic retinoid, acitretin, as the compound was previously shown to induce granulocytic differentiation of APL cells both *in vitro* and *in vivo* ^32^. Furthermore acitretin belongs to a group of retinoids, which induce differentiation but do not cause PML-RARα degradation^32^. Acitretin treatment for 6 days resulted in a strong upregulation of *ALFY* mRNA in control transduced NB4 cells (Fig. 4d). As observed in the case of ATRA, ALFY depletion significantly reduced acitretin-dependent induction of *CEBPE*, *CSF3R* and NBT-positivity (Fig. 4d–f). As we observed that ATRA causes a significant upregulation of ALFY in the non-APL cell line, HL60 (Fig. 1c), we further assessed granulocytic differentiation in ALFY-depleted HL60 cells. Similar to what was observed in NB4 cells, ALFY knock-down reduced the potential of HL60 cells to undergo granulocytic differentiation upon exposure to ATRA, as assessed by *CEBPE* and *CSF3R* mRNA levels (Fig. 4g). In conclusion, our data indicate that ALFY plays a crucial role in retinoid-triggered neutrophil differentiation of AML cells.

### NB4 ALFY knock-down cells display reduced proteolysis upon exposure to retinoids

**Figure 5:**
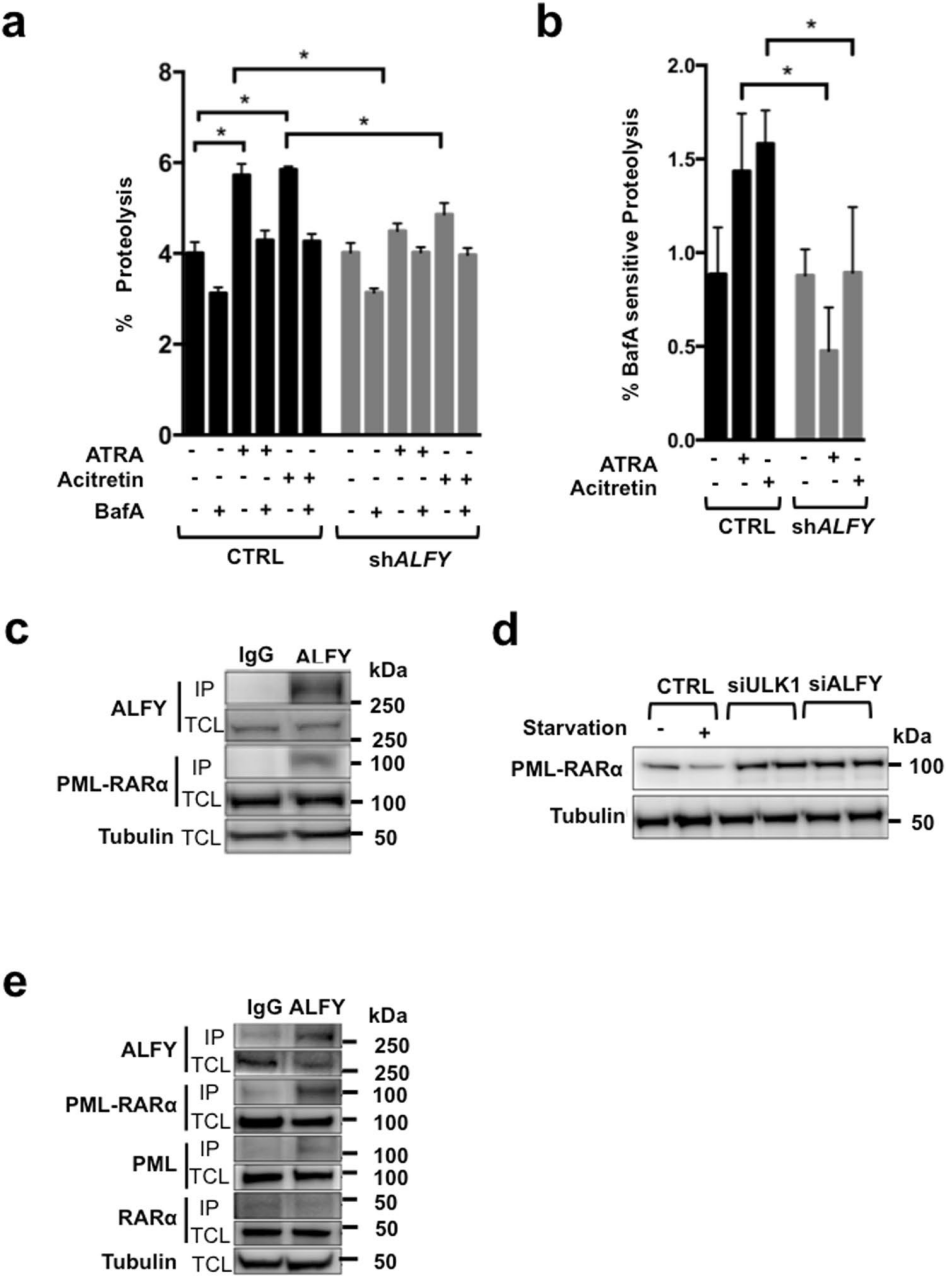
ALFY contributes to retinoid-induced lysosomal protein degradation. (**a**) Control and ALFY knockdown NB4 cells were treated with 1 µM ATRA or acitretin and long-lived protein degradation assays were carried out at day 4. Where indicated, bafilomycinA1 was added to inhibit lysosome dependent protein degradation. Radioactivity was determined by liquid scintillation counting of at least 3 independent experiments. The percentage of total proteolysis is shown. (**b**) The panel contains the data of the long-lived protein degradation assays as in A. The results are expressed as bafilomycinA1 (BafA) sensitive proteolysis which was calculated by subtracting the value of the BafA treated sample from the corresponding single agent treatment. (**c**) HEK293Tcells over-expressing PML-RARα were subjected to immunoprecipitation using anti-ALFY or the corresponding IgG antibody as a control. Western blot (WB) analysis of the immunoprecipitated proteins (IP) and of the total cell lysate (TCL) was performed using RARα (to detect PML-RARα at 110 kDa) and ALFY antibodies. Tubulin was used as a loading control. (**d**) HEK293T cells were transfected with control RNA, ULK1- or ALFY-targeted siRNA followed by transfection with PML-RARA cDNA. The different cell lines were subjected to starvation for 4 hours before western blot analysis of PML-RARα (~110 kDa) was performed. Tubulin is shown as a loading control. (**e**) The immunoprecipitation of NB4 cell lysates with anti-ALFY or the corresponding IgG antibody is shown. The Immunoprecipitated proteins (IP) and the total cell lysate (TCL) were subjected to western blot analysis for PML-RARα (110 kDa, using RARα antibody), RARα (~50 kDa) and ALFY using Tubulin as a loading control.

To get insights into the molecular and cellular processes underlying the action of ALFY on granulocytic differentiation we performed a number of studies in NB4 cells. As ALFY is known to mediate autophagy-dependent degradation of protein aggregates^22^, we measured lysosomal proteolysis under ATRA and acitretin treatment using the long-lived protein degradation assay (LLPDA). As expected ATRA^33^ as well as acitretin treated NB4 control cells displayed enhanced proteolysis of long-lived proteins (Fig. 5a). To measure lysosomal degradation, we used bafilomycinA1 (BafA), an inhibitor of the vacuolar type H^+^-ATPase. In fact, BafA inhibits the acidification of lysosomes, suppressing protein degradation^34^. In control transduced cells, BafA partially blocked ATRA- and acitretin induced proteolysis, confirming that the two retinoids induce protein degradation via the lysosomal compartment (Fig. 5a). In contrast, ATRA or acitretin treatment caused only a minor and non-significant increase of protein degradation in ALFY-silenced NB4 cells (Fig. 5a). Importantly, BafA-sensitive and thereby autophagy-dependent proteolysis was significantly lower in retinoid treated ALFY knock-down NB4 cells as compared to control transduced cells (Fig. 5b). Of note, basal proteolysis was not affected by ALFY knock-down. Together these results suggest a role for ALFY in retinoid-mediated proteolysis.

Since PML-RARα is degraded via autophagy in a p62 dependent manner^14^, we wondered whether ALFY is involved in this process. To this end, we used HEK293T cells overexpressing PML-RARα to avoid confounding factors generated by the differentiation process activated by ATRA in myeloid cells. Using this model we found ALFY in a complex with PML-RARα in co-immunoprecipitation experiments (Fig. 5c). Moreover, the levels of PML-RARα decreased upon induction of autophagy by serum and amino acid starvation (Fig. 5d). The starvation dependent down-regulation of PML-RARα was however not seen in cells depleted of the core autophagy protein ULK1, confirming its degradation by autophagy. Similarly, depletion of ALFY resulted in higher basal levels of PML-RARα that were not reduced upon starvation (Fig. 5d). ALFY also interacts with PML-RARα in NB4 cells, as endogenous PML-RARα, but not RARα, co-immunoprecipitated with endogenous ALFY (Fig. 5e). These results suggest that PML-RARα is a target for starvation-induced autophagic degradation when over-expressed in HEK293T cells and that ALFY is involved in this process. The participation of ALFY in endogenous PML-RARα degradation in NB4 cells needs further investigation, although it is clear that ALFY knock-down significantly reduces lysosomal degradation upon retinoid exposure.

## Discussion

In the present study we found that exposure of AML blasts to ATRA and a derived synthetic retinoid (acitretin) leads to increased expression of the autophagy related gene ALFY. In addition, the knock-down of ALFY decreases or delays the granulocytic differentiation of AML cells. Interestingly, *ALFY* mRNA was significantly up-regulated during G-CSF-triggered neutrophil differentiation of CD34^+^ progenitor cells. In accordance with this last finding, ALFY mRNA expression is increased in granulocytes as compared to neutrophil precursors and hematopoietic stem cells. Thus, our data provide evidence for the involvement of ALFY not only in the granulocytic differentiation of AML blasts, but also of normal myeloid cells. Importantly, we demonstrated lower levels of ALFY in AML patients than in mature granulocytes from healthy donors. The observation suggests that the low amounts of ALFY observed in AML are not simply due to deregulated expression of the gene in this disease but they rather reflect the immature myeloid phenotype of the leukemic blasts.

We further investigated how *ALFY* mRNA is regulated during the granulocytic differentiation of the APL-derived and PML-RARα positive NB4 cell line. In particular, we focused on the potential role of miRNAs in the control of ALFY expression. MiRNAs are small non-coding RNAs regulating mRNA expression. MiRNAs can be oncogenic or tumor suppressive and are often dysregulated in cancer^35^. A recent set of studies has implicated miRNAs in the regulation of autophagy including ATRA-triggered autophagy via targeting of mRNAs controlling different steps of the pathway^36,37^. Here we report that ALFY is yet another autophagy gene regulated by a miRNA in APL cells, i.e. miR-181b. Although we cannot exclude that miR-181b regulates *ALFY* mRNA indirectly, our data demonstrate that over-expression of miR-181b in NB4 cells leads to down-regulation of the *ALFY* transcript even under control conditions. The down-regulation of miR-181b in NB4 cells exposed to ATRA^30,31^ provides one explanation for the up-regulation of *ALFY* mRNA during neutrophil differentiation. Interestingly, members of the miR-181 family have been shown to attenuate autophagy via targeting ATG5 under various conditions^38,39^. As ATG5 inhibition exerts a negative impact on neutrophil differentiation of AML cells as well^14^, we propose that the marked inhibitory effects of miR-181b expression on neutrophil differentiation are due to its ability to target more than one ATG gene.

Autophagy is an important homeostatic process taking place in virtually all cells at basal levels that can be further upregulated under stress^40^. It is well established that autophagic flux is activated upon ATRA stimulation of APL cells^13,14,33,41,42^, where it contributes to the degradation of PML-RARα^13,14^. ALFY is an autophagy scaffold protein involved in the degradation of ubiqutinated protein aggregates^21,22^. In agreement with its function in aggrephagy, we found reduced autophagy-dependent proteolysis in retinoid treated ALFY knock-down cells. One potential substrate that is targeted to the autophagosome by ALFY is PML-RARα. Indeed, both ALFY and PML-RARα are part of a complex in HEK293T cells. Furthermore, starvation induced autophagic degradation of ectopic PML-RARα is ALFY dependent, and it is very likely that ALFY also contributes to therapy-induced degradation of PML-RARα in NB4 cells. Importantly, PML-RARα degradation is key to long-term APL remission as it reduces the number of LICs^32^. This emphasizes the importance of developing innovative therapies aiming at degrading PML-RARα. With respect to this, autophagy induction may represent a valid strategy for the treatment of APL since it contributes to PML-RARα degradation at least *in vitro* ^13,14^. Further studies aiming at defining the effects of autophagy induction on PML-RARα levels in LICs, the contribution of ALFY to the process and the impact on the therapeutic effect are of interest.

PML-RARα is unlikely to be the only substrate to be degraded with the assistance of ALFY as it could not fully explain the block in differentiation. In fact, PML-RARα degradation is not required for the granulocytic differentiation of APL blasts, as indicated by studies performed with uncoupling retinoids, a series of compounds which induce differentiation but do not degrade PML-RARα^32^. In addition, PML-RARα stabilization via reduction of its proteasomal degradation enhances the differentiation capacity of ATRA treated APL cells *in vitro* ^43,44^

Consistent with these last observations, we demonstrate that the uncoupling retinoid, acitretin, accelerates the maturation process of NB4 cells causing an induction of the neutrophil differentiation markers, *CEBPE* and *CSF3R*, as well as the up-regulation of ALFY. Noticeably, ALFY depletion has a negative impact on acitretin-dependent differentiation of NB4 cells. This supports the notion that ALFY controls the degradation of additional proteins during neutrophil differentiation. In line with this hypothesis, our data indicate that ALFY is also crucial for ATRA-triggered granulocytic differentiation of PML-RARα negative HL60 AML cells. Analyzing binding partners of ALFY during neutrophil differentiation of HL60 or CD34^+^ cells might allow identification of proteins in addition to PML-RARα that need to be degraded for effective differentiation.

During the process of myeloid differentiation and maturation, cells undergo major morphological and homeostatic changes to acquire novel and specific functions. Autophagy might contribute to this process due to its ability to degrade whole organelles as shown for late stage erythroid development where autophagy is involved in removing mitochondria^45,46^. In addition, autophagy plays a crucial role in cell survival during stress conditions by recycling cellular building blocks^47^. Therefore, autophagy could support differentiation by providing metabolic substrates. Alternatively it is involved in regulating signalling pathways by selective degradation of signalling molecules^48^. In this context, ALFY could support the degradation of cargo molecules involved in morphological changes or regulating protein signalling levels via aggrephagy. Indeed, ALFY participates in the control of Wnt signalling by removing aggregates of DVL3 via autophagy^49^. Clearly, additional studies are required to support all these hypotheses and to investigate possible non-autophagy related functions of ALFY which may provide information as to its role in AML differentiation.

## Materials and Methods

### Cell lines and culture conditions

HEK293T were obtained from American Type Culture Collection (ATCC). HEK293 cells were grown in Dulbecco modified Eagle medium (Invitrogen) containing 10% fetal bovine serum (Sigma-Aldrich). For starvation experiments, cells were incubated in Earle balanced salt solution (EBSS; Invitrogen) for 4 hours. The human acute promyelocytic leukemia (APL) cell line, NB4, its all-*trans* retinoic acid (ATRA)-resistant NB4-R2 clone as wells as ATRA-resistant HL60 cells were kindly provided by B.E.Torbett. HT93 and HL60 cells were purchased from DSMZ (http://www.dsmz.de). All cell lines were cultured in RPMI-1640 medium (Sigma-Aldrich, Buchs, Switzerland) supplemented with 10% foetal bovine serum (FBS), 50 U/ml penicillin and 50 µg/ml streptomycin in humidified atmosphere containing 5% CO_2_ at 37 °C. For the differentiation experiments, NB4, NB4-R2, HL60 and HL60-R2 cells were seeded at a density of 0.1 × 10^6^/ml and HT93 at 0.3 × 10^6^/ml and treated with 1 µM ATRA (Sigma-Aldrich, Buchs, Switzerland) or 1 µM acitretin (Sigma-Aldrich, Buchs, Switzerland) for indicated amounts of time. Successful neutrophil differentiation was assessed by increased CCAAT/enhancer binding protein ε (*CEBPE*) and colony stimulating factor 3 receptor (*CSF3R*) mRNA expression as described in section 4.3 or by NBT assays (refer to 4.4). CD34^+^ cells were isolated and differentiated *in vitro* towards granulocytes as described^50^.

### Generation of knock-down cells

For the combined transfection of HEK293T cells with plasmid and siRNAs, cells were first transfected with 40 nM single sequence control siRNA (D-001810–01–20, Dharmacon), siRNAs against human *ALFY* (J-012924-05, Dharmacon), ULK1 (J-005049-08-0005, Dharmacon) by the use of the Lipofectamine RNAiMax transfection reagent (Thermo Fisher Scientific). Three days later, cells were transfected with a plasmid encoding PML-RARA (a kind gift from Dr Pier Giuseppe Pelicci, Istituto Europeo di Oncologia) by the use of the FuGENE transfection reagent (Roche) and left for another 24 or 48 hours to perform immunofluorescence microscopy or Western blot analysis, respectively. NB4 cells were transduced with a lentiviral vector (pLKO.1) expressing small hairpin (sh)RNAs targeting *ALFY, PU.1* or *CEBPA* mRNA at different sites (*ALFY*: #1 = NM_178583.1-796s21c1, #2 = NM_178583.1-1696s21c1, #3 = NM_178583.1-1045s21c1) (*PU.1*: #1 = NM_003120.1-256s1c1, #2 = NM_003120.1-928s1c1; *CEBPA*: #1 = NM_004364.2-171s1c1, #2 = NM_004364.2-942s1c1) or a non-targeting shRNA control (SHCOO2). All the vectors were purchased from Sigma-Aldrich (Buchs, Switzerland). Lentiviral production and transduction of NB4 and HL60 cells were as described^51^. Two days after transduction, NB4 and HL60 cells were selected with 1.5 µg/ml Puromycin (Sigma-Aldrich, Buchs, Switzerland) for 2 days followed by selection with 0.5 µg/ml for another 5 days.

### TaqMan low density array (TLDA) and quantitative PCR (qPCR)

RNA extraction was performed using the miRCURY RNA Isolation Kit from Exiqon (Vedbaek, Denmark) according to the manufacturer’s instructions. RNA was reverse-transcribed using the masterscript kit purchased from 5prime (Hilden, Germany). Quantitative PCR analysis of *ALFY*, *CEBPE* and *CSF3R* mRNAs was performed using TaqMan® reagents and the StepOnePlus qPCR system (Applied Biosystems, Zug, Switzerland). Raw Ct values were normalized to *HMBS* and to the untreated control of day 4 or 6 (2^−∆∆CT^ method^52^). The gene Expression assays for *ALFY* (Hs00385595_m1), *CEBPE* (Hs00797944_s1) and *CSF3R* (Hs00357657_m1) were ordered at Applied Biosystems (Rotkreuz, Switzerland). The *HMBS* primers and probes have been described previously^53^. QPCR analysis of *ALFY* in patients and primary granulocytes was performed using TaqMan Low-Density Arrays (TLDA) using the ABI7500 qPCR assay system (Applied Biosystems, Zug, Switzerland). Raw Ct values were normalized to *HMBS* and ABL-1 (∆Ct). TLDA measurements as well as data analysis were performed as described^54^.

### NBT assay

Cells were stimulated for 10 minutes with 200ng/ml phorbol myristate acetate (PMA) in the presence of 1 mg/ml NBT. Both reagents were purchased from Sigma-Aldrich (Buchs, Switzerland). Cells were fixed using CytoSpin and counterstained with Safranin (0.25 g safranin/50 ml dissolved in 20% ethanol). Quantification of the NBT assays was performed by counting the percentage of NBT positive cells. Differences in colour intensity were not considered.

### Quantitative PCR of microRNAs

Isolation of microRNAs was performed using the miRCURY™ Isolation Kit from Exiqon (Vedbaek, Denmark). Expression levels were determined using the miRCURY LNA™ universal RT microRNA PCR kit (Exiqon, Vedbaek, Denmark) according to manufacturer’s instructions. Raw C_t_ values were calculated according to the 2^−∆∆CT^ method as described in^52^

### Primary patient samples

RNA was isolated from 95 patients with primary AML (FAB classification M0-M4). Patients were enrolled in and experimental protocols were approved by HOVON/SAKK (protocols −04, −04A, −29, and −42; available at www.hovon.nl) between 1987 and 2006^55–58^. All patients provided written informed consent in accordance with the Declaration of Helsinki and all methods were performed in accordance with the relevant guidelines and regulations. After informed consent was given, bone marrow aspirates or peripheral blood samples were taken at diagnosis. Patient data are summarized in Supplementary Table 1. Granulocytes from healthy donors were isolated using Polymorphprep^TM^ (Axon Lab, Baden-Dattwil, Switzerland) according to manufacturer’s instructions.

### Long-lived protein degradation assay

NB4 cells were seeded at a density of 0.1 × 10^6^/ml and treated with vehicle, 1 µM ATRA (Sigma-Aldrich, Buchs, Switzerland) or 1 µM acitretin (Sigma-Aldrich, Buchs, Switzerland) for two days before the addition of 0.2µCi of ^14^C-Valin/ml (L-(U-14-C)Valine, Code CFB.75, Amersham). Cells were incubated for another two days before the long-lived protein degradation assays were performed. Cells were washed and incubated for 1.5 h in the presence of 10 mM non-radioactive L-Valin before the 5 h chasing phase was started. Protein precipitations were carried using trichloracetic acid (at 10%) and phosphotunctic acid (at 1%). After incubation at 4 °C for 30 minutes samples were centrifuged at 600 rcf for 15 minutes. Radioactivity in the supernatant and the pellet fractions was determined using a scintillation counter (PerkinElmer). Proteolysis was calculated as the percentage of radioactivity in the supernatant.

### Luciferase assay

The activity of miR-181b on the 3′UTR of ALFY was tested using the luciferase assay kit LightSwitch from SwitchGear Genomics (California, USA) according to manufacturer’s instructions. Briefly, H1299 cells were seeded at a density of 20‘000 cells per 96-well. 24-hours later, cells were transfected using the Lipofectamine2000 reagent (Life Technologies, Zug, Switzerland). 24-hours following transfection, luminescence was measured using Tecan Reader Infinite200 (Tecan, Switzerland). MicroRNA mimics or scrambled sequence were purchased from Qiagen (Hombrechtikon, Switzerland).

### Western blotting

Cells were lysed in urea buffer (8 M Urea, 0.5% Triton-X) and loaded on a 4–20% Criterion TGX precast gel (BioRad). Protein transfer was performed using Criterion Blotter system (BioRad). The following primary antibodies were used for western blotting; ALFY^21^; RARα (sc551, Santa Cruz); PML (sc966, Santa Cruz); ULK1 (8054, Cell Signaling); a-tubulin (5168, Sigma). The antibodies were diluted in 5% BSA TBS-0.1% Tween and incubated at 4 °C over-night. HRP-conjugated donkey anti-rabbit and donkey anti-mouse secondary antibodies (Jackson ImmunoResearch Laboratories) were diluted in 5% Milk TBS-0.1% Tween and incubated for 1 h at room temperature. Image acquisition was performed with a Syngene chemiluminescence imaging system (Syngene).

### Co-Immunoprecipitation experiments

Protein lysates used for IP were prepared from HEK293T or NB4 cells. Cells were harvested and lysed in ice cold NP40 buffer (125 mM KAc, 25 mM Hepes at pH 7.2, 5 mM EDTA, 2.5 mM MgAc, 0.05% NP40 and 1 mM DTT) containing a protease and phosphatase inhibitor cocktail (Roche, 04906845001). The cell lysates were used for IP with anti-ALFY serum or pre-immune serum^21^ at 4 °C for 10 to 12 h. After addition of protein A agarose beads (Amersham Biosciences, 17–0963–03) and protein G agarose beads (Amersham Biosciences, 17–0618–01), the incubation was continued for 1 h. Immunoprecipitates were washed three times with ice-cold lysis buffer and eluted with SDS loading buffer at 95 °C for 5 min.

## Electronic supplementary material

Supplementary Information
