# Supplementary material for: The autophagy scaffold protein ALFY is critical for the granulocytic differentiation of AML cells
Source: Sci Rep. 2017 Oct 11;7:12980. doi: 10.1038/s41598-017-12734-4 (PMC5636880; doi:10.1038/s41598-017-12734-4)

The autophagy scaffold protein ALFY is critical for the granulocytic differentiation of AML cells

Anna M. Schläfli[1,2], Pauline Isakson[3], E. Garattini[4], Anne Simonsen[5], and Mario P. Tschan[1,2]

[1]Division of Experimental Pathology, Institute of Pathology, University of Bern, Switzerland, [2]Graduate School for Cellular and Biomedical Sciences, University of Bern, Switzerland, [3]Clinical immunology & transfusion medicine, Sahlgrenska University Hospital, Gothenburg, Sweden, [4]Laboratory of Molecular Biology, Istituto di Ricerche Farmacologiche 'Mario Negri', Milano, Italy, and [5]Department of Molecular Medicine, Institute for Basic Medical Sciences, University of Oslo, Norway.

Table 1. AML patient characteristics from the HOVON/SAKK cohort where ALFY mRNA levels were determined

		Patient characteristics			FAB classification					Cytogenetics				
**Cohort**	**Variables**	Age (y)	Sex (female/male)	**Total**	M0	M1	M2	M3	M4	t(8;21)	inv (16)	t(15;17)	CK	NK
HOVON/ SAKK	**Range**	18-74												
	**Mean/median or %**	43.06/43.00 (mean/median)	58.95/41.05	**100**	4.21	15.79	32.63	20	27.36	21.05	16.84	21.05	23.16	17.89
	**No. of patients**		56/39	**95**	4	15	31	19	26	20	16	20	22	17

FAB, French-American-British; **CK**, complex karyotype; **NK**, normal karyotype

Supplementary figure legends

Figure S1 Determination of knock-down efficiency and differentiation capacity of NB4 cells silenced for *PU.1* or *CEBPA* (a) Two different NB4 *PU.1* knock-down cell lines (#1 and #2) and control transduced cells were treated with 1 μM ATRA for 4 days. Thereafter qPCR analysis of *PU.1* and *CEBPE* mRNA was performed. Raw Ct values were normalized to *HMBS* and to the untreated control of day 4 ($2^{-\Delta\Delta CT}$ method). Mann-Whitney-U test: *P<0.05. (b) Same experiment as in a for two NB4 *CEBPA* silenced cell lines and the corresponding control cells.

Figure S2 ALFY depleted HT93 APL cell lines are impaired in ATRA-dependent differentiation. HT93 ALFY knock-down cell lines (#1 and #2) and control transduced cells were treated with 1 μM ATRA for 4 days. QPCR analysis of *ALFY*, *CEBPE* and *CSF3R* mRNA was performed and raw Ct values were normalized to *HMBS* and to the untreated control of day 4 ($2^{-\Delta\Delta CT}$ method). Mann-Whitney-U test: *P<0.05, **P≤0.01, ***P≤0.001.

Figure S3 ALFY and ULK1 knock-down efficiency in siULK1 and siALFY treated HEK293T cells. HEK293T cells were transfected with control RNA, ULK1- or ALFY-targeted siRNA followed by transfection with PML-RARA cDNA. Knock-down efficiency of ULK1 and ALFY was determined by western blot. α-Tubulin is shown as a loading control.

Supplementary figure 1

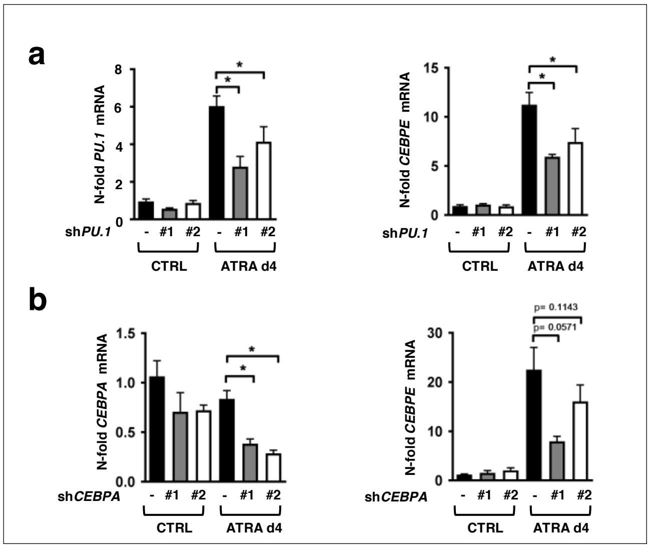

Supplementary figure 2

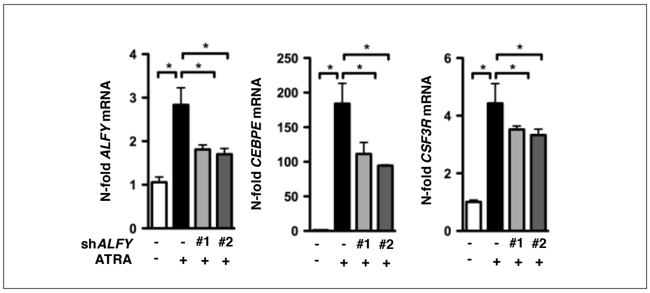

Supplementary figure 3

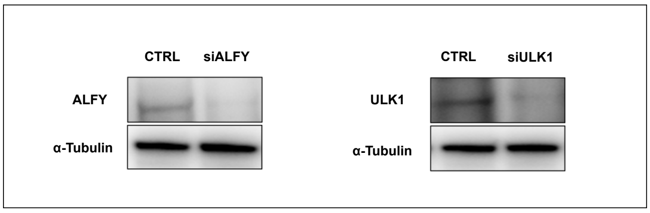

Supplement: Supplementary file 1 — Supplementary Information [file 41598_2017_12734_MOESM1_ESM.pdf]
